# Oncolytic Newcastle disease virus activation of the innate immune response and priming of antitumor adaptive responses in vitro

**DOI:** 10.1007/s00262-020-02495-x

**Published:** 2020-02-22

**Authors:** Shannon Burke, Amy Shergold, Matthew J. Elder, Justine Whitworth, Xing Cheng, Hong Jin, Robert W. Wilkinson, James Harper, Danielle K. Carroll

**Affiliations:** 1 Oncology R&D, AstraZeneca, Aaron Klug Building, Granta Park, Cambridge, CB21 6GH UK; 2 BioPharmaceuticals R&D, Clinical Pharmacology & Safety Sciences, AstraZeneca, South San Francisco, CA USA; 3 Present Address: Meissa Vaccines, JLABS, 329 Oyster Point Boulevard, 3rd Floor, South San Francisco, CA USA

**Keywords:** Newcastle disease virus, Oncolytic virus, Immunotherapy, MEDI5395

## Abstract

Oncolytic virus (OV) therapy is an emerging approach with the potential to redefine treatment options across a range of cancer indications and in patients who remain resistant to existing standards of care, including immuno-oncology (IO) drugs. MEDI5395, a recombinant Newcastle disease virus (NDV), engineered to express granulocyte–macrophage colony-stimulating factor (GM-CSF), exhibits potent oncolytic activity. It was hypothesized that activation of immune cells by MEDI5395, coupled with its oncolytic activity, would enhance the priming of antitumor immunity. Using MEDI5395 and recombinant NDVs encoding fluorescent reporter genes, we demonstrated preferential virus uptake and non-productive infection in myeloid cells, including monocytes, macrophages, and dendritic cells (DCs). Infection resulted in immune-cell activation, with upregulation of cell surface activation markers (e.g., CD80, PD-L1, HLA-DR) and secretion of proinflammatory cytokines (IFN-α2a, IL-6, IL-8, TNF-α). Interestingly, in vitro M2-polarized macrophages were more permissive to virus infection than were M1-polarized macrophages. In a co-culture system, infected myeloid cells were effective virus vectors and mediated the transfer of infectious NDV particles to tumor cells, resulting in cell death. Furthermore, NDV-infected DCs stimulated greater proliferation of allogeneic T cells than uninfected DCs. Antigens released after NDV-induced tumor cell lysis were cross-presented by DCs and drove activation of tumor antigen-specific autologous T cells. MEDI5395 therefore exhibited potent immunostimulatory activity and an ability to enhance antigen-specific T-cell priming. This, coupled with its tumor-selective oncolytic capacity, underscores the promise of MEDI5395 as a multimodal therapeutic, with potential to both enhance current responding patient populations and elicit de novo responses in resistant patients.

**Electronic supplementary material:**

The online version of this article (10.1007/s00262-020-02495-x) contains supplementary material, which is available to authorized users.

## Introduction

Despite advances in targeted therapies (small molecules and antibodies) and, more recently, IO drugs such as immune checkpoint inhibitors, cancer remains a leading cause of mortality [1]. A significant proportion of patients whose disease remains unresponsive or develops resistance to current IO therapies underscores the need for novel therapeutic approaches. Oncolytic viruses, a diverse class of viruses broadly characterized historically by their tumor cell-killing potential, have re-emerged as a promising option for cancer therapy with the recent clinical approval of a modified herpesvirus, talimogene laherparepvec (TVEC), for malignant melanoma [2]. Driving this is the realization that OVs demonstrate an ability to activate the immune system coincident with tumor cell killing. As such, OVs are increasingly considered to be multimodal IO agents whose antitumor activity could be further enhanced by combination with other IO or cancer therapeutics. Advances in virus engineering and understanding of tumor biology have enabled the development of multiple OVs exploiting different cellular mechanisms to drive tumor selectivity, oncolytic efficacy, and immunogenicity. Indeed, several other candidates are currently being evaluated in the clinic, including the reovirus, Reolysin [3] and coxsackievirus A21, CVA21 (Cavatak) [4], for which interim results suggest some antitumor efficacy with manageable safety profiles. The approval of TVEC in patients with metastatic melanoma and the recent demonstration that its use could potentiate current IO therapy provide a further rationale for development of other OV candidates [^5^]. However, limitations of the current OV strategies, such as their restriction to intratumoral delivery, indicate the need to develop OVs with broader applicability.

In parallel with the development of new-generation OV approaches, our understanding of why some patients respond to IO-based therapies and others remain resistant has also evolved. Responding tumors are often associated with an immunologically ‘hot’ phenotype, characterized by a high mutational burden and high density T-cell infiltrate. Conversely, ‘cold’ tumors contain increased suppressive immune-cell populations (e.g., myeloid-derived suppressive cells and T regulatory cells) and a low density of T-cell infiltrate and exhibit a low mutational burden and/or antigen expression [^6^]. The multimodal mechanisms of OVs make them well suited to transform the tumor microenvironment (TME) of non-responding patients into ‘hot’ tumors; the liberation of tumor antigens via immunogenic cell lysis is expected to increase T-cell infiltration and, by way of their immunogenic properties, may also potentially reverse established suppressive elements [7]. Additionally, these same properties are expected to enhance responses in those patients who already benefited from IO therapy and, as such, provide rationale for MEDI5395 and IO combination approaches.

Newcastle disease virus (NDV) is a negative-strand RNA avian paramyxovirus that exhibits both potent oncolytic activity and immunostimulatory properties [8]. We previously described MEDI5395, an attenuated form of the wild-type 73-T strain of NDV [9]. NDV elicits a strong type I interferon (IFN) response via engagement of cytosolic retinoic acid-inducible gene I (RIG-I)-like RNA innate immune sensors [10, 11]. Through virus engineering, MEDI5395 immune modulatory potential has been further enhanced by insertion of a transgene encoding granulocyte–macrophage colony-stimulating factor (GM-CSF) [9]. The resulting therapeutic candidate, MEDI5395, maintains NDV’s potent oncolytic activity against a wide range of human tumor cells (Harper et al., manuscript in preparation); however, its immunostimulatory properties have not been fully explored. It was hypothesized that, coincident with virus-induced tumor killing, MEDI5395 would elicit a type I IFN response together with other proinflammatory cytokines in the TME and initiate the generation of a robust systemic adaptive antitumor immune response.

The effect of MEDI5395 on primary human immune-cell populations that may impact the antitumor immune response is described here. Exposure of human immune cells to MEDI5395 led to preferential, but non-productive, infection of cells within the myeloid lineage, including monocytes, macrophages, and dendritic cells (DCs). However, unlike tumor cell infection, this did not result in efficient virus replication, but to type I IFN and pro-inflammatory cytokine production and enhanced antigen presentation and T-cell activation. Furthermore, infected myeloid cells could transfer MEDI5395 to uninfected tumor cells, leading to tumor cell lysis. Tumor-derived antigens released during NDV infection were efficiently taken up by DCs and were presented in a stimulatory context to tumor antigen-specific T cells. These data support the hypothesis that, in addition to its direct tumor-killing activity, MEDI5395 has potent immunostimulatory effects that have the potential to transform the TME (e.g., from ‘cold’ to ‘hot’) and enhance antitumor immunity.

## Materials and methods

### Virus infections and measurement of infectious particles

NDV was prepared and purified as described previously [9]. Virus infections were initiated by incubating cells at the indicated multiplicity of infection (MOI) at 37 °C and 5% CO_2_. In some cases, virus was incubated with the cells, followed by three washes of complete medium before the cells were returned to the incubator. Infectious virus titers were measured with a plaque assay. Briefly, tenfold serial dilutions of virus stock or assay samples were placed on Vero cells (American Type Culture Collection, Manassas, VA, USA) and incubated in 2% methylcellulose in complete medium for 6 days. Chicken anti-NDV antibody (Abcam, Cambridge, UK) was added in blocking solution for 1 h at room temperature. Plates were washed, the secondary antibody was applied [rabbit anti-chicken Y (H + L)-horseradish peroxidase] and incubated for 1 h, plates were washed again, and 3-amino-9-ethylcarbazole substrate was added for 45 min. Plates were washed and then allowed to dry before plaques were counted and virus titers were calculated.

### Cell lines

Tumor cell lines used in this study were cultured according to standard procedures. HT10180 fibrosarcoma cells were maintained in minimum essential medium plus 10% fetal calf serum (FCS; Thermo Fisher Scientific, Waltham, MA, USA), and MEL624 melanoma and MDA-MB-231 breast cancer cells lines were maintained in Dulbecco’s modified Eagle medium plus 10% FCS. For production of MEL624 and MDA-MB-231 cells expressing the pp65 protein of human cytomegalovirus, cells were transduced with lentivirus expressing the full-length coding sequence of pp65 (Atum, Newark, CA, USA) and a puromycin resistance cassette. Successfully transduced cells were selected in the presence of 0.5-mg/mL puromycin (Thermo Fisher Scientific) before use in the indicated assays.

### PBMCs and cell isolation

Peripheral blood mononuclear cells (PBMCs) from leukocyte cones from healthy donors were isolated with standard Ficoll-Paque Plus (GE Healthcare, Chicago, IL, USA) density gradient centrifugation. PBMCs from donors with known human leukocyte antigen (HLA) type and antigen reactivity were purchased from Cellular Technology Limited (OH, USA). Whole PBMCs were resuspended in culture medium (RPMI-1640, 10% FCS, 1% penicillin–streptomycin solution; all from Thermo Fisher Scientific) and cultured at 37 °C and 5% CO_2_ for the indicated time. CD14-expressing (CD14^+^) cells, plasmacytoid DCs (pDCs), and T cells from PBMCs were isolated by using CD14^+^ positive selection, pDC isolation, and CD3^+^ negative selection kits (StemCell Technologies, Cambridge, MA, USA), respectively, with a RoboSep automated cell separator (StemCell Technologies).

### Monocyte-derived macrophage and moDC differentiation

For differentiation of macrophages and monocyte-derived DCs (moDCs), CD14^+^ monocytes were resuspended in culture medium supplemented with 100-ng/mL macrophage colony-stimulating factor (M-CSF) (PeproTech, Rocky Hill, NJ, USA) and cultured at 37 °C and 5% CO_2_ for 6 days. Macrophage cultures were supplemented on day 3 with another 100-ng/mL M-CSF. Day 6 macrophages were gently scraped from the culture plates after brief treatment with Accutase (Thermo Fisher Scientific), washed with phosphate-buffered saline (PBS), counted, and re-plated in fresh medium to adhere overnight in appropriate assay plates. Polarization of macrophages was conducted according to standard protocols, using IFN-γ (M1) or interleukin-4 (IL-4) and IL-13 (M2) (PeproTech, Rocky Hill, NJ, USA) [12]. Immature moDCs were derived from CD14^+^ monocytes by culturing at 37 °C and 5% CO_2_ for 6 days in medium supplemented with 100-ng/mL GM-CSF and 100-ng/mL IL-4 (both from PeproTech). moDC cultures were supplemented on day 3 with another 100-ng/mL GM-CSF and 100-ng/mL IL-4. Floating moDCs were collected and the loosely adherent cells were recovered with gentle scraping. Both fractions were pooled and plated in appropriate assay plates. In some cases, differentiated cells were infected with virus at the indicated MOI and washed before re-plating.

### T-cell assays

For Staphylococcal enterotoxin B (SEB) stimulations, flat-bottomed plates were coated with 0.5-µg/mL anti-CD3 (OKT3; BioLegend, San Diego, CA, USA), and SEB (Sigma Chemical Company, St. Louis, MO, USA) was added at a concentration of 450 ng/mL. Whole PBMCs were added with the indicated dose of virus and incubated for 3–5 days before the supernatant was harvested. For DC–T-cell mixed leukocyte reactions and DC presentation assays, isolated T cells and differentiated moDCs were co-cultured in U-bottomed plates at the indicated ratio and incubated at 37 °C and 5% CO_2_. For DC presentation assays, immature moDCs were, where indicated, first matured by the addition of lipopolysaccharide (LPS; Sigma Chemical Company) (10 ng/mL) and IFN-γ (100 IU/mL) [13] and cultured overnight. Mature moDCs were then washed thoroughly and co-cultured with T cells. In the indicated experiments, 2.5 μM HLA-A*0201-restricted CMV-specific peptide, pp65 363–373 (NLVPMVATV), was added. The cultures were maintained for 5 days before the supernatant was harvested.

### Cell staining and flow cytometry

After collection, cells were washed in PBS and resuspended in ultraviolet (UV) live/dead Zombie dye (BioLegend) and labeled according to the manufacturer’s instructions. Cells were then washed with fluorescent activated cell sorting (FACS) buffer (PBS, 1% bovine serum albumin, 0.1% NaN_3_) and resuspended in TruStain FcX (BioLegend) for 15 min to block Fc receptors before the addition of specific antibodies at prevalidated concentrations. Antibodies were obtained from BioLegend, Thermo Fisher Scientific, or Abcam (Cambridge, UK). Cells were incubated with antibodies for a further 30 min and then washed twice with FACS buffer. After staining, cells were fixed with 4% formalin and resuspended in FACS buffer. Samples were acquired with a LSRFortessa flow cytometer (BD Biosciences, Franklin Lakes, NJ, USA), and data were analyzed with FlowJo software (FlowJo, Ashland, OR, USA). Gating was determined with fluorescence-minus-one, unstained, and untreated controls. For labeling of macrophages before co-culture with tumor cells, CellTracker Orange CMTMR dye (Thermo Fisher Scientific) was used according to the manufacturer’s instructions. Apoptosis of tumor cell targets was measured by the addition of IncuCyte caspase 3/7 reagent (Essen BioScience, Ann Arbor, MI, USA) to the cell culture before imaging with IncuCyte ZOOM (Essen BioScience).

### Cytokine measurement

The secreted cytokines were measured in cell-free cell culture supernatants, using the IFN-α2a kit, GM-CSF kit, and Human Proinflammatory panel Meso Scale Discovery platform (Meso Scale Diagnostics, Rockville, MD. USA), or validated IFN-γ (BD BioSciences), IL-2 and CCL13 (R&D Systems, Minneapolis, MN, USA) enzyme-linked immunosorbent assay (ELISA) protocols according to the manufacturers’ directions. Standard curves and results were generated with Discovery workbench software (Meso Scale Diagnostics) for the Meso Scale Discovery kits and Prism software program, version 7.04 (GraphPad Software, La Jolla, CA, USA), for ELISAs. Enzyme-linked immunosorbent spot assays (ELISPOTs) were performed with 96-well ELISPOT plates (Millipore Sigma, Billerica, MA, USA) and human IFN-γ ELISPOT antibody kits (Mabtech, Cincinnati, OH, USA). Spots were enumerated with an ImmunoSpot plate reader (Cellular Technology Limited Europe, Bonn, Germany).

### Statistics

Prism software, version 7.04, was used for determination of statistical significance. A two-way analysis of variance was applied to the data in the original scale, considering treatment and dose as two different factors, and the *P* value of the interaction was assessed. Dunnett’s test was applied for multiple comparisons in case of a significant *P* value. Significance was reported as *P < 0.05; **P < 0.01; and ***P < 0.001.

## Results

### Proinflammatory response to MEDI5395 in PBMCs

**Fig. 1:**
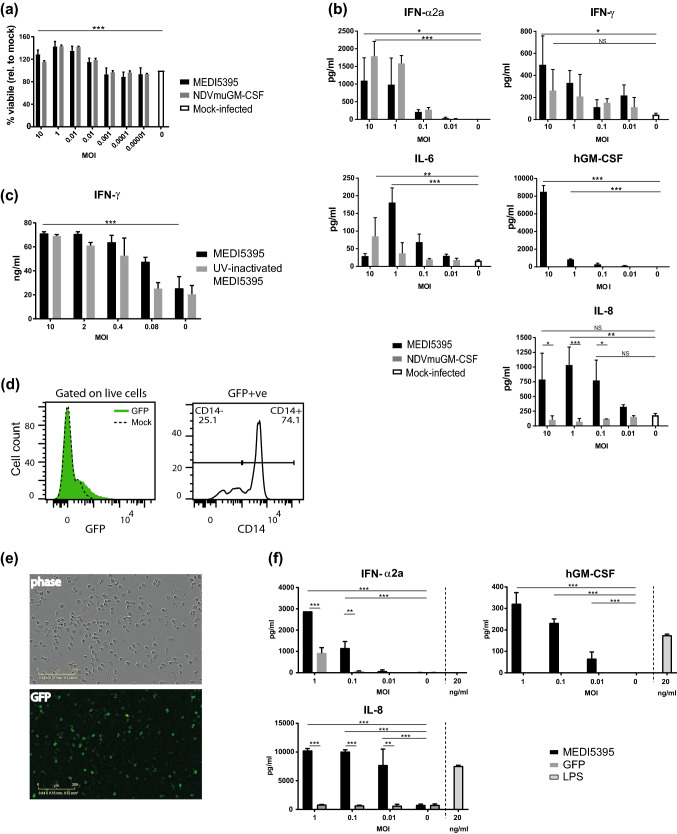
Infection of whole PBMCs with NDV. Whole PBMCs prepared from the blood of healthy donors were infected with a dose titration of the indicated purified virus. **a** Viability at 24 h of PBMC cultures relative to mock-infected controls (M). **b** After 24 h of incubation, cell-free supernatants were collected and analyzed for proinflammatory cytokines. **c** IFN-γ response of whole PBMCs to SEB stimulation in the presence of the indicated dose of virus or UV-inactivated virus. **d** Flow cytometric analysis of whole PBMCs incubated for 24 h with NDV-GFP at MOI 1. Live cells were gated to examine GFP expression (left panel) and the proportion of GFP^+^ cells expressing CD14^+^ (right panel). **e**, **f** CD14^+^ cells were enriched from whole PBMCs, plated, and treated with the indicated dose of virus or LPS. GFP expression was monitored over time (**e**; MOI 1), and supernatants were collected and analyzed for proinflammatory cytokines (**f**). Results are representative data from two (**c**, **e**, **f**), three (**a**, **d**), and five (**b**) independent experiments, each of which used cells from at least two donors. Data shown in bars are mean ± standard error of the mean. *P < 0.05, **P < 0.01, ***P < 0.001 compared to mock-infected (MOI 0) controls

To investigate the immunomodulatory activity of NDV, PBMCs isolated from healthy human donors were infected with MEDI5395. A surrogate NDV expressing murine GM-CSF (NDVmuGM-CSF), which does not cross-react with the human GM-CSF (hGM-CSF) receptor [14], was used to assess the contribution of the therapeutic GM-CSF transgene in this setting. After 24 h of incubation with virus, cell viability, as measured by ATP release, had increased at higher doses of MEDI5395 (approximately 20% increase at MOI 10; *P* < 0.001) compared with media-treated (mock-infected) controls, suggesting that virus infection had stimulated cell proliferation (Fig. 1a). There was no difference in this phenotype between the viruses, demonstrating that this effect was virus mediated and not impacted by transgene expression. Cell-free supernatants were analyzed for proinflammatory cytokines as an indicator of immune-cell activation. Typical of RNA viruses, both MEDI5395 and NDVmuGM-CSF infection induced high levels of type I IFN, IFN-α2a, production compared with mock-infected controls (*P* = 0.03 and *P* < 0.001, respectively, at MOI 10; Fig. 1b). Levels of the T-cell and natural killer (NK) cell effector cytokine IFN-γ also increased in a dose-dependent manner after infection with MEDI5395 (*P* = 0.02 at MOI 10). An increase in IFN-γ release was observed after NDVmuGM-CSF infection but did not reach statistical significance. IL-6 production was also significantly increased after MEDI5395 (*P* < 0.001 at MOI 1) infection compared with mock-infected controls. That PBMC cultures supported transgene expression was demonstrated by a significant increase in hGM-CSF production compared with mock-infected controls (*P* < 0.001 at MOI 10). Because this assay could not differentiate between NDV-encoded GM-CSF expression or that produced by immune cells after NDV stimulation, NDVmuGM-CSF virus was used as a control. Mouse GM-CSF was detected only in those cultures infected with NDVmuGM-CSF, where it increased significantly in a dose-dependent manner (*P* < 0.001 at MOI 10; Supplemental Fig. S1a). IL-8 production, which can in part be regulated by GM-CSF [15], was also significantly upregulated by MEDI5395, but not NDVmuGM-CSF, infection at all but the lowest MOI (0.01) tested (*P* = 0.003 at MOI 10), indicating that hGM-CSF produced by infected cells was bioactive. This demonstrated that the production of hGM-CSF was solely dependent on the expression of transgene from infected cells.

Stimulation of PBMCs by MEDI5395 infection led to the activation of specific leukocyte effector populations, as measured by the increased expression of CD69 on both T cells and NK cells (Supplemental Fig. S1b). Enhancement of T-cell function was further demonstrated in the context of the polyclonal T-cell mitogen SEB, where addition of MEDI5395 strongly potentiated IFN-γ production compared with the addition of medium alone (*P* < 0.001 at MOI 10) (Fig. 1c). A similar response was observed with UV-inactivated MEDI5395, demonstrating that virus replication was not required for T-cell activation in this context.

To further characterize the susceptibility profile of immune cells to NDV infection, PBMCs were incubated with an NDV encoding green fluorescent protein (NDV-GFP). Flow cytometry analysis at 24 h after infection revealed that only a small fraction (< 5%) of the total PBMC pool was GFP^+^ (Fig. 1d). Immune phenotyping of PBMCs showed that the majority (> 70%) of GFP^+^ cells were CD14^+^, suggesting infection of, and low-level replication in, cells of the myeloid lineage. The majority of the remaining GFP^+^ cells were identified as NK cells (CD3^–^ CD56^+^) and, to a lesser extent, T cells (CD3^+^ CD56^–^) (Supplemental Fig. S1c). B cells were GFP^–^ (data not shown). Despite evidence of GFP expression in PBMC subsets, no increase in virus titer was observed over 48 h (data not shown), indicating non-productive replication within individual immune-cell populations.

### Activation of innate immunity after MEDI5395 infection

Further investigation revealed that isolated CD14^+^ blood monocytes became infected when incubated with MEDI5395 or NDV-GFP (Fig. 1e). MEDI5395 infection induced high levels of IFN-α2a, hGM-CSF, and IL-8 in monocyte cultures compared with mock-infected controls (*P* < 0.001 for each at MOI 1; Fig. 1f). Interestingly, the IFN-α2a response was significantly greater in the monocyte cultures infected with MEDI5395 than in those infected with NDV-GFP (*P* < 0.001 at MOI 1), suggesting an additional stimulatory effect on monocyte cytokine production by MEDI5395-derived hGM-CSF that was not detected in whole PBMC cultures. Indeed, TNF-α and IL-6 were also significantly induced by MEDI5395 (*P* < 0.001 for both at MOI 1), but not by NDV-GFP, although the absolute levels of these cytokines remained relatively low (Supplemental Fig. S1d).

**Fig. 2:**
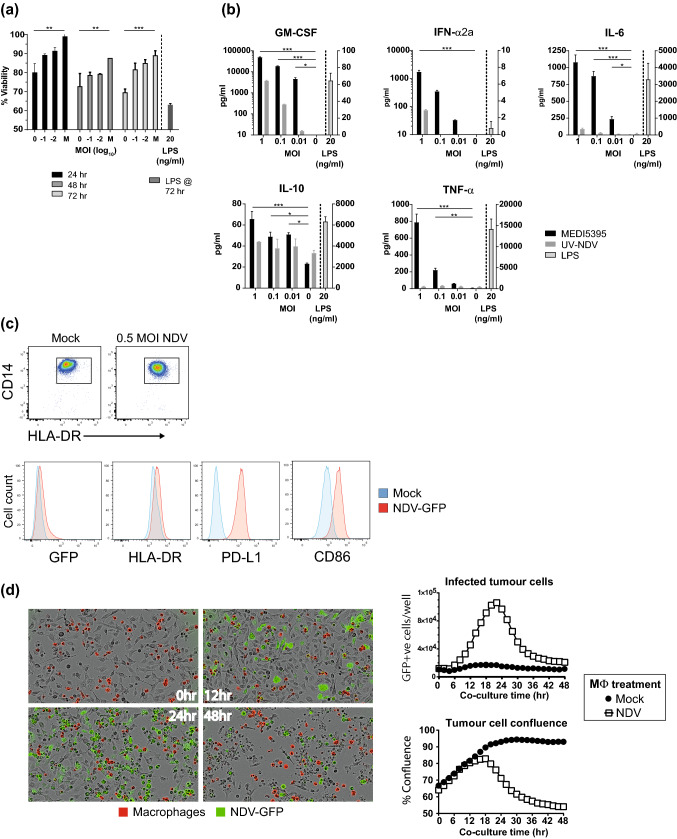
Infection and activation of macrophages by NDV and transfer of infectious virus to tumor cells. CD14^+^ cells were enriched from whole PBMCs and differentiated into monocyte-derived macrophage in the presence of M-CSF for 6 days. Cells were then re-plated before being infected with a dose titration of the indicated purified virus. **a** Viability of macrophages relative to mock-infected controls (M) over 72 h of culture. **b** After 24 h of incubation with virus or LPS, cell-free supernatants were collected and analyzed for proinflammatory cytokines. **c** Flow cytometric analysis of CD14^+^ macrophages (top) for GFP expression and the indicated phenotypic markers after mock infection and NDV-GFP infection at MOI 0.5 (bottom). Numbers in plots indicate gate percentage. **d** After infection with NDV-GFP at MOI 1, CMTMR-labeled macrophages were overlaid onto adherent HT1080 fibrosarcoma cells and monitored over 48 h with live imaging. Representative images from the indicated times after co-culture (left) and quantitation of tumor cell infection and tumor cell confluency (right). Results are representative data from two (**a**, **b**, **d**) and three (**c**) independent experiments, each of which used cells from two donors. Data shown in bars are mean ± standard error of the mean. *P < 0.05, **P < 0.01, ***P < 0.001 compared to mock-infected (MOI 0) controls

Next, the potential of NDV to directly infect macrophages or DCs was investigated. A low level of GFP expression, indicative of infection, was observed in macrophages and peaked at 12–18 h after infection (Supplemental Fig. S1e). This was accompanied by a dose-dependent decrease in cellular viability over time (*P* < 0.001 at MOI 1 by 72 h; Fig. 2a). Moreover, there was no detectable increase in infectious virus titer in the supernatants of these cultures (data not shown) above that of the level of input virus, indicating that infection was non-productive. However, infection was accompanied by the release of significantly enhanced levels of proinflammatory cytokines, including IFN-α2a, TNF-α, IL-6, and GM-CSF (*P* < 0.001 for each at MOI 1 compared with mock-infected controls; Fig. 2b). These functional changes were accompanied by the modulation of surface markers associated with activation (Fig. 2c and Supplemental Table S1). Upregulation of PD-L1 and CD86 was observed after infection (18.5- and 4.2-fold in mean fluorescence intensity compared with mock-infected cells, respectively), whereas HLA-DR, which was relatively highly expressed on uninfected cells, was only modestly upregulated (1.4-fold).

### Preferential infection of M2-polarized macrophages and delivery to tumor cells

To model the potential spectrum of tumor-associated macrophage populations, M0 macrophages were polarized to M1 (classically activated) and M2 (alternatively activated) states [12]. Analysis of supernatants following LPS stimulation for typical M1 (IL-12p70) and M2 (IL-10, CCL13) cytokines/chemokines confirmed their polarization (Supplemental Fig. S2a). After infection, only M2 and unpolarized M0 macrophages supported transgene expression (Supplemental Fig. S2b). M1 macrophages remained GFP^–^, even at high MOI. This pattern of infection was also reflected in the cytokine profiles of infected cells; IFN-α2a, IL-6, and TNF-α production was significantly enhanced 24 h after infection of M0 and M2 macrophages and was only modestly enhanced in the M1 population (Supplemental Fig. S2c).

To determine whether infected macrophages could act as carriers of NDV, fluorescently labeled monocyte-derived macrophages were infected with NDV-GFP, washed repeatedly, and then co-cultured with HT1080 fibrosarcoma cells. The co-cultures were imaged at 2-h intervals for 48 h to determine the degree of tumor cell killing. Consistent with the ability of free virus to infect and kill HT1080 cells, the number of GFP^+^ tumor cells increased in wells containing infected macrophages and peaked at 24 h (Fig. 2d), suggesting the transfer of NDV to the underlying tumor cell layer and the initiation of productive infection. This was followed by loss of tumor cell confluence and cell death by 48 h. Mock-infected macrophages had no discernible cytotoxic effect on HT1080 cells. Comparable results were also observed with DU145, a prostate cancer cell line (data not shown). These results indicate that infected macrophages can act as a vector and transfer virus that can subsequently replicate in and lyse susceptible tumor cells. Furthermore, these data suggest that transfer of NDV to tumor cells from a cell-associated form may be an additional alternative delivery mechanism, either as a clinical strategy analogous to that reported for reovirus [16] or as a positive by-product of in vivo monocyte/macrophage infection after virus administration.

### MEDI5395 induction of DC maturation and T-cell priming against tumor cell-associated antigens

**Fig. 3:**
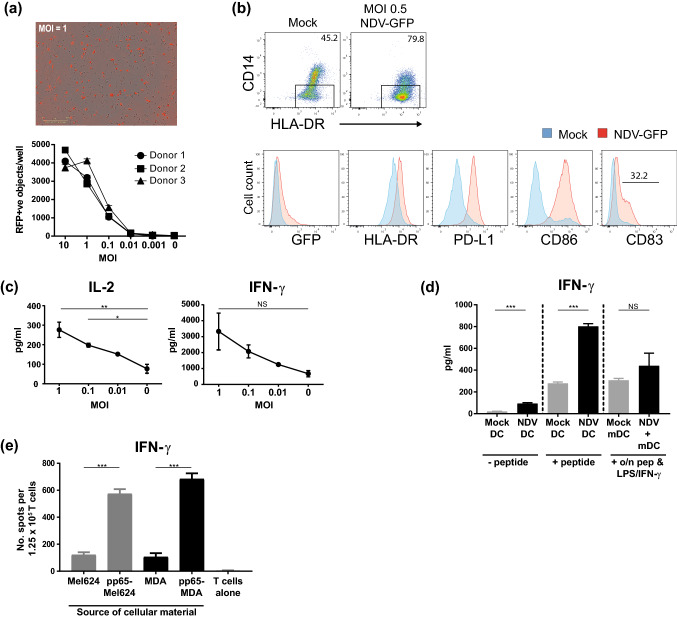
Induction of DC maturation by MEDI5395 infection. CD14^+^ cells were enriched from whole PBMCs and differentiated into monocyte-derived dendritic cells with GM-CSF and IL-4 for 6 days. Cells were then re-plated before infection with a dose titration of the indicated virus. **a** Representative images of infected moDCs at 24 h after infection with NDV-RFP at MOI 1 (top) and quantitation for moDC derived from three individual donors (bottom) across the indicated MOI of virus and mock-infected control (M). **b** Flow cytometric analysis of CD14^–^ moDCs (gated; top) for GFP expression and the indicated phenotypic markers after mock infection and infection with NDV-GFP at MOI 0.5 (bottom). Numbers in plots indicate gate percentage. **c** After infection with the indicated dose of virus, moDCs were plated with enriched allogeneic T cells and the IL-2 and IFN-γ response was analyzed at days 3 and 5 of culture, respectively. **d** Mock- or NDV-infected moDCs derived from characterized donors were pulsed with pp65 peptide or left untreated and were then co-cultured with enriched autologous T cells. Positive controls were mature moDCs that had been cultured overnight in the presence of LPS, IFN-γ, and pp65 peptide and then washed. The IFN-γ response was analyzed at day 5. **e** Immature moDCs were loaded for 48 h with apoptotic material from infected Mel624 or MDA-MB-231 cells or their pp65-expressing derivatives (pp65-Mel624, pp65-MDA), matured further with LPS, and co-cultured with enriched autologous T cells. The IFN-γ response was analyzed by ELISPOT at day 2 of co-culture. Results are representative data from two (**d**, **e**) and four (**a**–**c**) independent experiments, each of which used different donors (except **d** and **e**, in which one donor was used). Data shown in bars are mean ± standard error of the mean. *P < 0.05, **P < 0.01, ***P < 0.001 compared to mock-infected (MOI 0) controls (**c**) or between respective groups (**d**, **e**)

DCs are necessary for the initiation of antitumor immune responses and are therefore considered important for the efficacy of many IO therapies, including oncolytic virus therapy [17]. To examine the impact of NDV infection on DC activation and function, immature moDCs were generated by differentiating CD14^+^-enriched cells isolated from PBMCs for 6 days in the presence of GM-CSF and IL-4 and were subsequently infected with an NDV encoding red fluorescent protein (NDV-RFP). NDV replication within moDC cells was evident within 5–6 h of infection and peaked at 18 h (data not shown). A dose-dependent increase in the number of moDCs infected was observed (Fig. 3a). Interestingly, there was an approximately twofold increase in the proportion of CD14^–^ cells from NDV-infected cultures compared with mock-infected cultures, suggesting that NDV infection either drove the downregulation of CD14 expression or selectively killed CD14^+^ cells in moDC cultures (Fig. 3b). Expression of the DC marker CD209 was maintained on DCs infected with NDV (data not shown), suggesting maintenance of the DC lineage. To further examine phenotypic changes of moDC after infection with NDV, the modulation of surface markers associated with DC maturation was analyzed by flow cytometry 24 h after infection. NDV infection caused the upregulation of HLA-DR, PD-L1, and CD86 (2.4-, 7.4-, and 15.9-fold increases in mean fluorescence intensity compared with mock-infected cells, respectively; Fig. 3b and Supplemental Table S2). The DC activation molecule CD83 was also upregulated. Together, these data demonstrate that NDV infection of moDCs led to their activation, suggesting a possible mechanism by which MEDI5395 therapy may help to initiate or enhance a DC-mediated antitumor immune response.

pDCs are an important producer of type I IFN in response to RNA viruses [17]. Given that one of the proposed mechanisms by which NDV could enhance the antitumor immune response is via the stimulation of a strong type I IFN response, the cytokine output from freshly isolated pDCs infected with NDV-GFP was investigated. Similar to monocytes, pDC infection by NDV-GFP led to a low level of GFP expression, indicating limited virus replication (Supplemental Fig. S3a). This coincided with elevated levels of IFN-α2a and other proinflammatory cytokines IL-8, TNF-α, and IL-6 (Supplemental Fig. S3b), supporting the hypothesis that an NDV-infected TME could elicit a type I IFN-driven proinflammatory response that may in turn enhance an antitumor immune response.

The functional implications of NDV-induced DC maturation were assessed by using a DC–T-cell mixed leukocyte reaction. moDCs infected with MEDI5395 were co-cultured with allogeneic T cells and assessed for levels of the T-cell effector cytokines IL-2 and IFN-γ as a measure of the capacity of infected DCs to stimulate T cells. DCs infected with MEDI5395 induced increased allogeneic T-cell activation compared with mock-infected DCs, as evidenced by a dose-dependent increase in the levels of IL-2 (3.4-fold at MOI 1; *P* < 0.001) and IFN-γ (4.9-fold at MOI 1; not significant) (Fig. 3c).

To demonstrate this function in an antigen-specific context, autologous PBMC preparations (Cellular Technology Limited, OH, USA) from donors with validated recall responses to a known HLA-A*02 CD8^+^ T-cell epitope from the pp65 protein of human cytomegalovirus were tested. Immature moDCs from these PBMCs were prepared and either infected with MEDI5395 or mock infected. They were then cultured with enriched T cells from the same donor in the presence or absence of pp65 peptide. In the absence of pp65 peptide, MEDI5395 infection induced low levels of IFN-γ, whereas the response to mock-infected moDCs was almost undetectable (Fig. 3d). In the presence of exogenous pp65 peptide, MEDI5395 infection potentiated IFN-γ production in these cultures (greater than twofold increase compared with mock-infected moDCs; *P* < 0.001) (Fig. 3d), consistent with the finding that NDV caused the maturation of moDCs. Interestingly, comparison of this response with positive control DCs (matured in the presence of peptide and LPS/IFN-γ) [13] suggests that NDV-induced DC maturation was highly efficient. Together these data demonstrate that infection of DC populations with MEDI5395 resulted in their functional activation, enhancing their ability to stimulate T cells in both allogeneic and autologous settings.

One proposed mechanism by which MEDI5395 infection is likely to enhance the antitumor immune response is by mediating the release of tumor-derived antigens after lysis of infected cells, which could then be taken up by antigen-presenting cells, including DCs, and presented to T cells. To test this hypothesis, MEDI5395-sensitive tumor cell lines, MEL624 and MDA-MB-231, were transduced with a lentivirus expressing full-length pp65 protein to provide a traceable tumor-derived antigen. After infection and subsequent lysis of these cells or the respective parental tumor lines by NDV-RFP, the resulting apoptotic material was incubated for 48 h with moDC (Supplemental Fig. S3c). The material was not taken from mock-infected tumor cells, as no significant cell death was observed. DCs were then co-cultured with autologous pp65-reactive CD3^+^ T cells and the IFN-γ response was detected by ELISPOT. Significantly greater cytokine release was observed from T cells in response to DCs precultured with NDV-lysed pp65-expressing cells than from parental tumor cell controls (greater than threefold increase in both Mel624-pp65 and MDA-MB-231-pp65 cells, *P* < 0.001 for both; Fig. 3e). These findings demonstrate that tumor-derived antigens released during NDV infection were efficiently taken up by DCs and presented in a stimulatory context to specific T cells.

## Discussion

OV therapy has the potential to transform treatment in many cancer settings. Previously, we have described efforts to develop an OV with a multimodal mechanism of action, including broad oncolytic activity and an ability to enhance the antitumor immune response [9]. Here, the impact of MEDI5395 infection on human immune-cell populations was examined. It has been demonstrated that NDV induces activation of immune-cell populations, including human PBMCs [18], NK cells [19, 20], and mouse macrophages [21-24]. These findings are extended here with a comprehensive analysis of human PBMC populations acting as surrogates for subsets of immune cells present within the TME and the peripheral circulation. MEDI5395 infection of PBMCs elicited a robust proinflammatory cytokine response, including bioactive GM-CSF transgene expression, self-limiting infection of myeloid populations, and concomitant activation of specific immune-cell populations, including NK cells, macrophages, and DCs. These observations support the hypothesis that MEDI5395 drives an immune phenotype consistent with the positive transformation of the TME [6].

MEDI5395 is engineered to express a GM-CSF transgene, a cytokine with pleiotropic immunomodulatory properties, including the ability to enhance aspects of antigen presentation within the TME [25]. In whole-PBMC preparations, MEDI5395 was found to induce human immune-cell activation independently of GM-CSF transgene expression, whereas GM-CSF produced from NDV replication was found to further enhance monocyte activation and IL-8 production. These results suggest that the effect of the GM-CSF transgene is dependent on the cellular context and may act directly on monocyte-derived cells such as DCs and macrophages [25]. These observations demonstrate the potential of including immune-modulating transgenes in the development of NDV-based therapeutics to further enhance antitumor activity.

The data presented here inform our understanding of the possible impact of MEDI5395 infection on the TME. A potent type I IFN and proinflammatory cytokine response was observed after NDV infection, consistent with engagement of the cytosolic virus sensing proteins RIG-I and toll-like receptor 3 (TLR-3) and TLR-7/8 [26]. Although RIG-I is expressed in most cell types, TLR-3 and TLR-7 expression is restricted to DCs and other antigen-presenting cells that are present in the TME and are believed to play a critical role in the recognition of cancer by the immune system [27]. Immune cells in the TME would probably be activated through these pathways. In many cancers, the tumor cell-intrinsic type I IFN response is believed to be deficient [4]. In such cases, triggering of a type I IFN response via the immune component of the TME may benefit the antitumor immune response. IO therapies with an immune-activating profile, such as demonstrated here with the phenotypic and functional activation of innate immune cells, are proposed to contribute to the favorable transformation of so-called “cold” tumors (those with an immune composition associated with poor prognosis, including low T-cell infiltrate) to “hot” tumors [6, 28].

The finding that moDCs could endocytose MEDI5395-infected tumor cells and present tumor-derived antigens to specific T cells supports a mechanistic link between the noted oncolytic activity of NDV and its ability to promote antitumor immune responses. NDV-induced tumor cell lysis, which may involve direct induction of proapoptotic signals via RIG-I engagement [29], would release antigens and cellular debris from susceptible tumors, thus contributing to the initiation of tumor-specific immune responses. Although a model antigen (pp65) was used in the present study, it is conceivable that therapy-induced tumor destruction in vivo may allow for the presentation of novel tumor-specific antigens, thus broadening the antitumor T-cell response, through the process of antigen spreading [30]. Moreover, presentation of tumor antigens by local DCs and some macrophage populations would probably be rendered immunogenic by the type I IFN-driven inflammation associated with infection. Indeed, IFN-α itself is known to augment DC cross-presentation [31]. The observation that direct infection of DCs by MEDI5395, a possible by-product of therapy, enhanced their function as antigen-presenting cells suggests a further mechanism by which MEDI5395 may impact the priming of antitumor immune T-cell response. A similar DC-maturing effect has been observed using an NDV-GFP virus [32]. Concomitant to this, release of IFN-γ by activated T cells, as observed in vitro here, may upregulate PD-L1 expression within the TME. Indeed, the combination of OVs and PD-1 blocking antibodies are already being testing in the clinic [28].

Several cancer types are characterized by different tumor-associated macrophage populations [33]. M2-polarized macrophages, though not a direct surrogate of tumor-associated macrophages or myeloid-derived suppressive populations, are clearly distinguished from classically activated antitumoral macrophages. MEDI5395 selectively infected M2- but not M1-polarized macrophages and augmented their cytokine output, suggesting a mechanism through which MEDI5395 may reduce the immunosuppressive potential within the TME. In support of this hypothesis, others have demonstrated that type I IFN signaling is linked to the reprogramming of myeloid-derived suppressor cells [34, 35]. This finding may help guide patient or indication selection for MEDI5395 therapy to those where strong M2 signatures within the TME are observed.

The ability for any OV to remain in the circulation long enough to achieve its maximal therapeutic potential before neutralization by antiviral antibodies remains a key challenge for OV therapy development, particularly where there is pre-existing immunity to the virus vector. Consequently, many OV strains are limited to intratumoral delivery. NDV has advantages over most OV platforms in this regard because humans are not natural hosts and, being rarely exposed, have little pre-existing immunity [8]. The wild-type NDV strains PV701 and HUJ have been shown in phase 1/2 clinical trials to be amenable to intravenous and systemic delivery, to be well tolerated, and to elicit some objective responses [36, 37]. The findings presented here showing that NDV differentially infected cells of the myeloid lineage and that macrophages could act as a vector to mediate infection of tumor cells suggest a potential mechanism by which intravenous MEDI5395 may transit to tumor lesions. Circulating monocytes that become infected with MEDI5395 may contribute to this effect upon egress from the blood and differentiation into macrophages or DCs within the TME. These observations, together with our previous data demonstrating efficient tumor targeting after intravenous delivery of MEDI5395 in preclinical mouse models [9], provide a strong rationale for the clinical development of intravenously delivered, NDV-based OV therapies.

The emerging portfolio of cancer drugs, such as immune checkpoint blockade monoclonal antibodies and antibody–drug conjugates, has been providing much-needed therapeutic options and has afforded a small proportion of patients with unprecedented levels of survival compared with the current standards of care. However, as a large proportion of treated patients have disease that remains unresponsive or later develops resistance, new therapeutic strategies are urgently required. Guided by the hypothesis that successful treatment of diverse cancer indications will require an agent exhibiting multiple mechanisms of action, we have developed MEDI5395, a recombinant NDV exhibiting several beneficial attributes. The results presented here provide evidence that MEDI5395 would promote immune-cell activation and proinflammatory responses and/or reduce the immunosuppressive milieu dominant in many cancers. Translated to the human setting, these observations have the potential to provide a new benchmark in the standards of care across a range of cancer indications and to improve overall patient survival.

### Electronic supplementary material

Below is the link to the electronic supplementary material.

Supplementary file1 (DOCX 2.57 mb)

## Abbreviations

hGM-CSF: Human granulocyte–macrophage colony-stimulating factor
IO: Immuno-oncology
IV: Intravenous
M-CSF: Macrophage colony-stimulating factor
moDC: Monocyte-derived dendritic cell
MOI: Multiplicity of infection
NDV: Newcastle disease virus
NDVmuGM-CSF: Newcastle disease virus murine granulocyte–macrophage colony-stimulating factor
NK cell: Natural killer cell
OV: Oncolytic virus
pDC: Plasmacytoid dendritic cell
RFP: Red fluorescent protein
RIG: Retinoic acid-inducible gene
SEB: Staphylococcal enterotoxin B
