# Supplementary material for: Oncolytic Newcastle disease virus activation of the innate immune response and priming of antitumor adaptive responses in vitro
Source: Cancer Immunol Immunother. 2020 Feb 22;69(6):1015–27. doi: 10.1007/s00262-020-02495-x (PMC7230062; doi:10.1007/s00262-020-02495-x)
Supplement: Supplementary file 1 — Supplementary file1 (DOCX 2.57 mb) [file 262_2020_2495_MOESM1_ESM.docx]

**SUPPLEMENTARY TABLES AND FIGURES**

**Supplementary Table S1** Cell surface marker expression on human monocyte–derived macrophages after infection with MEDI5395

| **Experiment and donor** | **MOI** | **Cell surface marker expression^a^** | | |
| --- | --- | --- | --- | --- |
|  |  | **HLA-DR** | **PD-L1** | **CD86** |
| Experiment 1  Donor 1  Donor 2 | 0  0.5  **Δ**  0  0.5  **Δ** | 2491  3544  **1.4**  3619  6638  **1.8** | 85.3  1585  **18.5**  117  1844  **15.7** | 971  4083  **4.2**  725  6490  **9.0** |
| Experiment 2  Donor 1  Donor 2 | 0  0.1  **Δ**  0  0.1  **Δ** | 1353  1795  **1.3**  1056  1777  **1.7** | 101  2145  **21.2**  161  1601  **9.9** | 436  1606  **3.6**  608  1549  **2.5** |

^a^Expression level was determined as the mean fluorescence intensity (MFI) of the test antibody relative to the isotype or unstained control. Δ = Fold-change expression (in bold type) was determined by dividing the MFI of infected cells by the MFI of mock-infected cells at multiplicity of infection (MOI) 0.

**Supplementary Table S2** Cell surface marker expression on human monocyte–derived dendritic cells after infection with MEDI5395

| **Experiment and donor** | **MOI** | **Cell surface marker expression** | | | |
| --- | --- | --- | --- | --- | --- |
|  |  | **HLA-DR^a^** | **PD-L1^a^** | **CD86^a^** | **% CD83^b^** |
| Experiment 1  Donor 1  Donor 2 | 0  1  0  1 | ND  ND  ND  ND | ND  ND  ND  ND | ND  ND  ND  ND | 3.9  68.1  7.97  91.9 |
| Experiment 2  Donor 1 | 0  1  **Δ** | ND  ND  ND | ND  ND  ND | 517  16115  **31.2** | 3.4  86.2  ND |
| Experiment 3  Donor 1  Donor 2 | 0  0.5  **Δ**  0  0.5  **Δ** | 4667  11177  **2.4**  5693  18866  **3.3** | 326  2409  **7.4**  432  3480  **8.0** | 337  5370  **15.9**  207  9483  **45.8** | 3.9  32.2  ND  6.32  54.5  ND |

^a^Expression level was determined as MFI of the test antibody relative to the isotype or unstained control. Δ = Fold-change expression (in bold) was determined by dividing the MFI of infected cells by the MFI of mock-infected (MOI 0) cells. ^b^Percentage CD83^+^. ND = not determined.


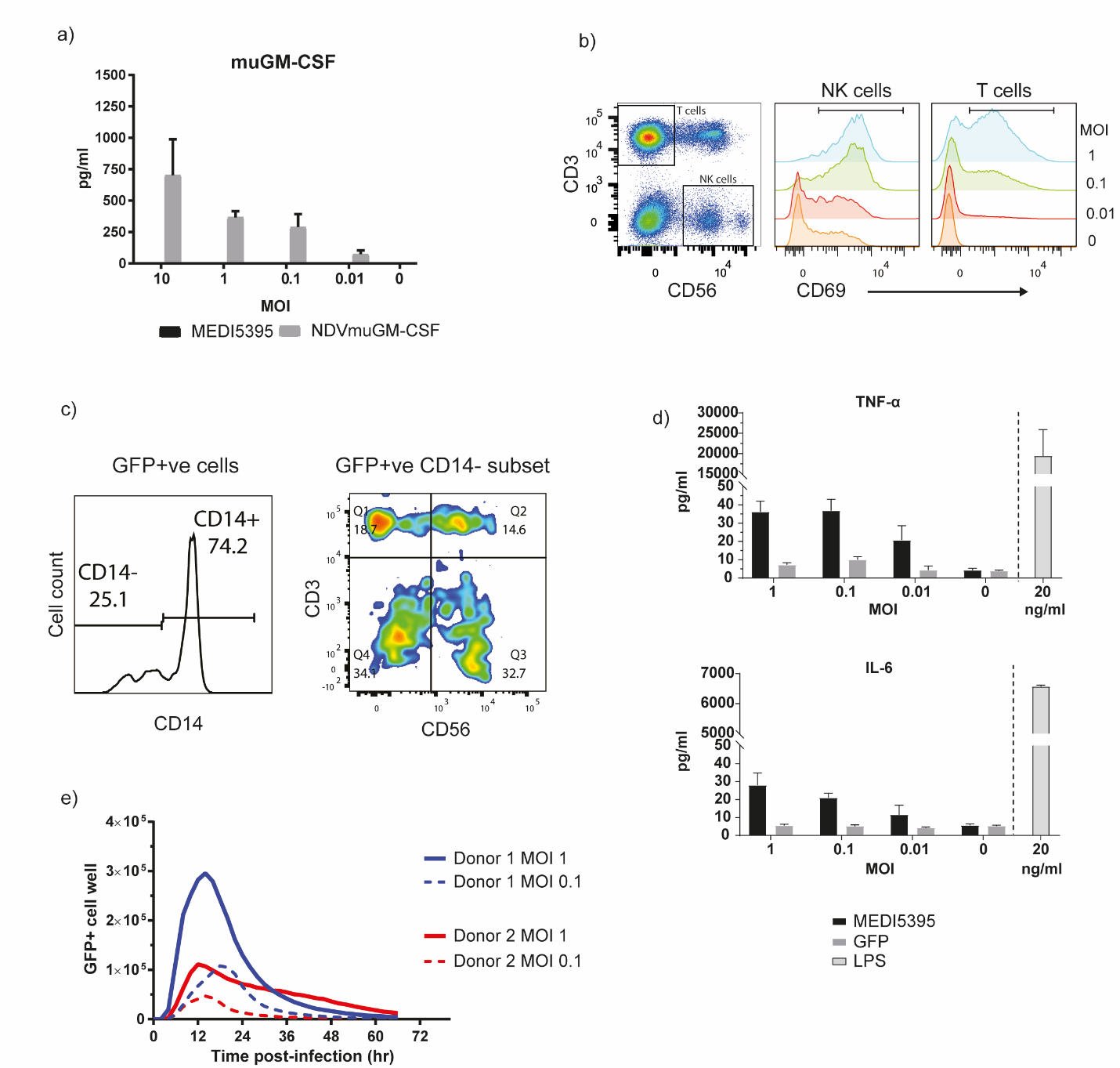


**Supplementary Fig S1** **Infection and activation of human leukocytes following exposure to Newcastle disease virus (NDV).** PMBCs from healthy donors were infected with a dose titration of the indicated virus and incubated. After 24 hours, cell-free supernatants were collected and analyzed for proinflammatory cytokines. **a** Levels of murine GM-CSF (muGM-CSF) in supernatants of PBMC cultures infected with MEDI5395 or Newcastle disease virus (NDV) muGM-CSF. Data are mean ± SEM of triplicate wells and are representative of at least four independent donors. **b** Flow cytometry analysis of NK and T cell (gated, left) CD69 expression at 24 hours after infection with MEDI5395. Marker indicates CD69^+^ cells. Data are representative of two independent donors. **c** Flow cytometry analysis of CD14 expression on live GFP^+^ PBMCs (left) and expression of CD3 and CD56 on the CD14^-^ subset (right) after infection with NDV-GFP. Numbers in gates are percentages. **d** CD14^+^ monocytes infected at the indicated MOI of MEDI5395 or NDV-GFP were treated with LPS and supernatants were analyzed for the indicated cytokine at 24 hours after infection. **e** Monocyte-derived macrophages from two independent donors were infected with NDV-GFP at the indicated MOI and followed by live imaging.


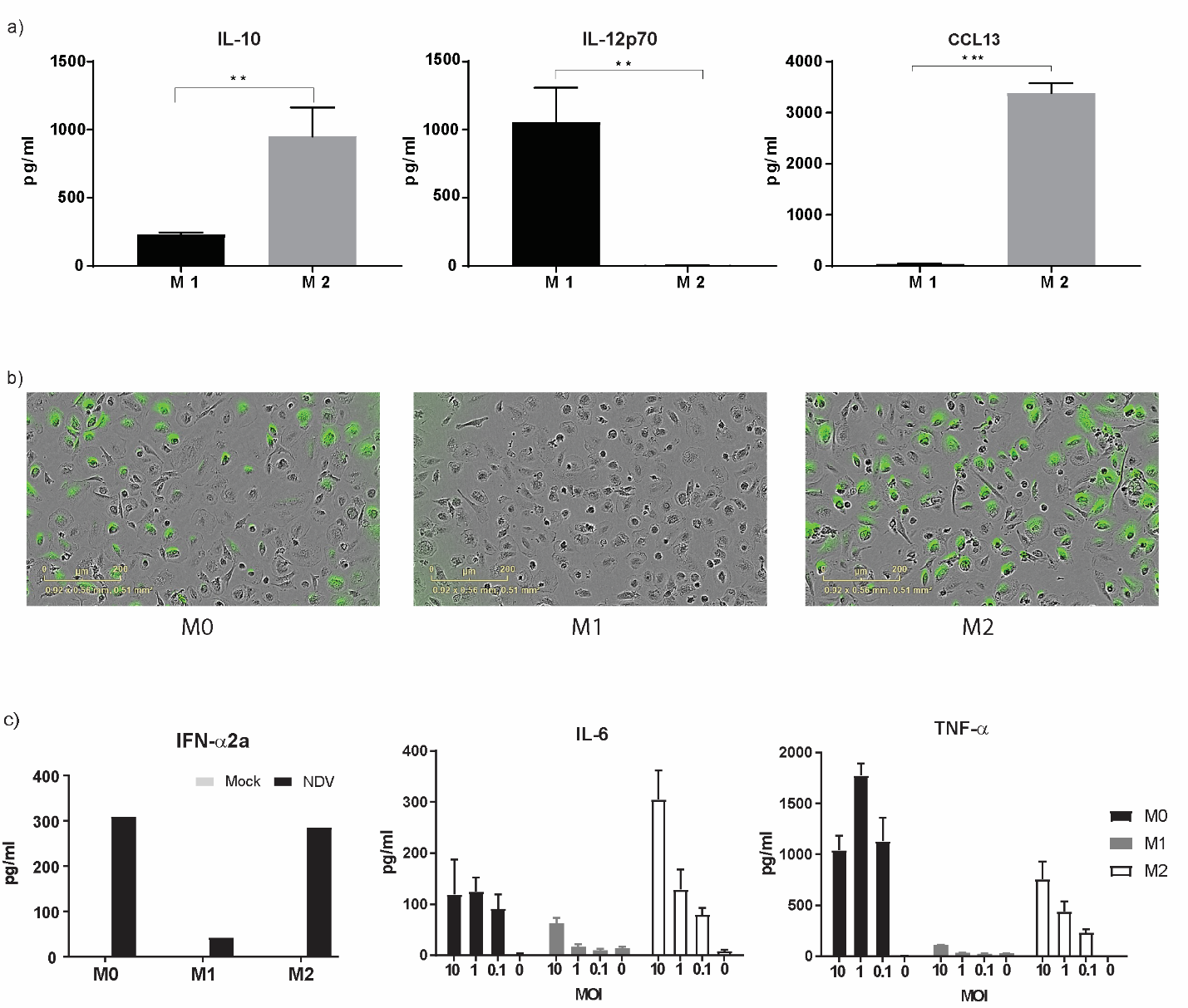


**Supplementary Fig S2 Infection and activation of in vitro-derived human macrophages.** CD14^+^ cells were enriched from whole PBMCs and differentiated to monocyte-derived macrophage in the presence of macrophage CSF (M-CSF) for 6 days. Cells were re-plated before being polarized to M1 or M2 macrophages or were left unpolarized (M0). **a** Polarised macrophages were stimulated with 20 ng/ml LPS and supernatants were analysed for the indicated M1 (IL-12p70) and M2 (IL-10, CCL13) signature cytokines at 24 hr. **b** Cells were then infected with NDV-GFP at MOI 1 and imaged using Incucyte. Representative fields from live imaging of polarized macrophages showing GFP image overlaid on bright field. **c** Supernatants from these cultures were collected and analyzed for cytokines.

**
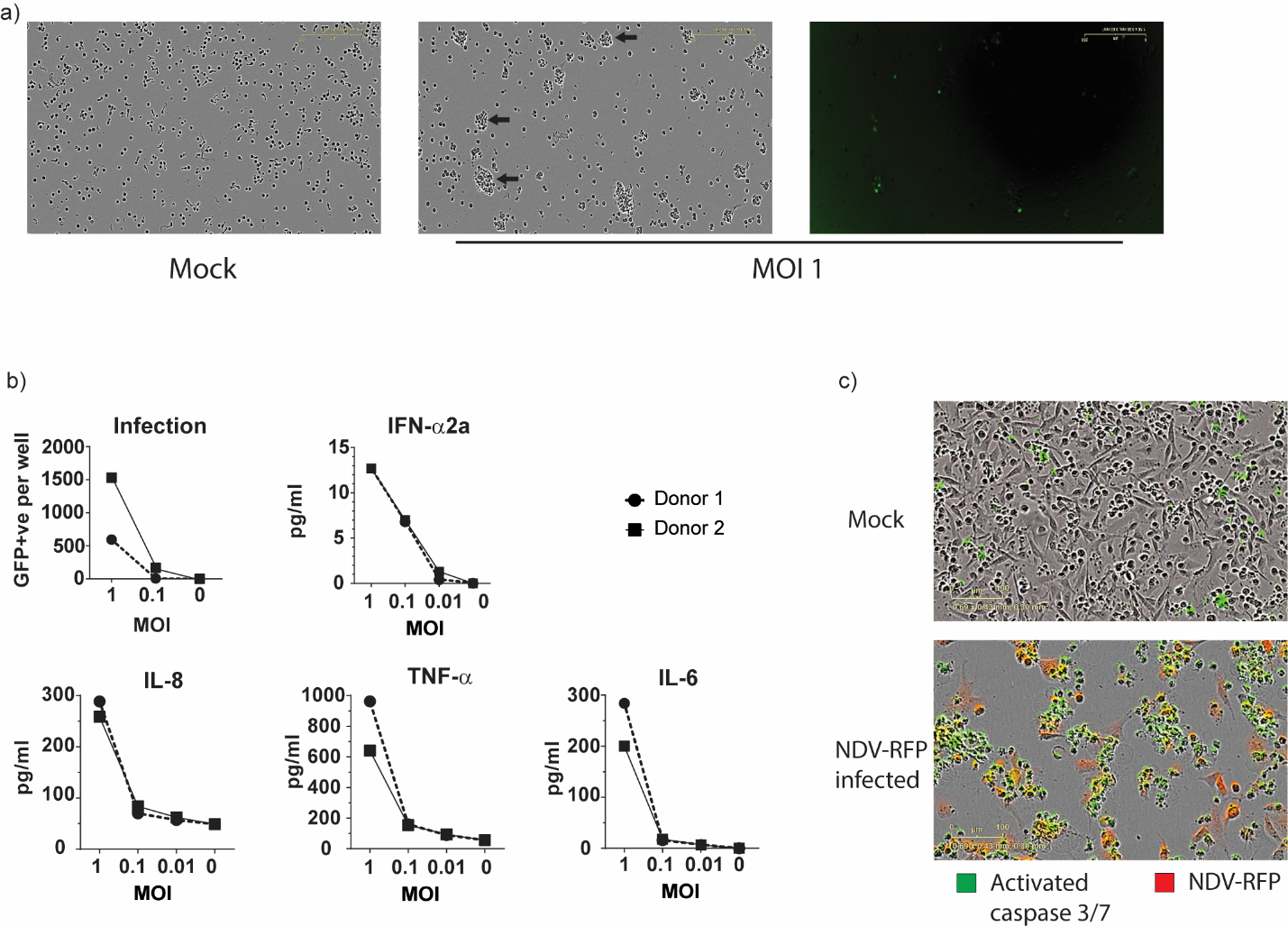
**

**Supplementary Fig S3 Activation of plasmacytoid dendritic cells by MEDI5395 and evidence of tumor cell apoptosis following NDV infection.** **a** Plasmacytoid dendritic cells (pDCs) were isolated and infected with the indicated dose of NDV-GFP. After 24 hours of incubation, cell-free supernatants were collected and analyzed for proinflammatory cytokines. The left panel shows mock-infected pDCs, and the middle and right panels show infected pDCs with clustering evident (arrows). **b** Quantification of GFP^+^ cells per well and cytokine analysis from two individual donors. **c** MDA-MB-231 tumor cells were mock infected (top) or infected with NDV encoding red fluorescent protein (NDV-RFP) at MOI 5 (bottom) and cultured for 48–36 hours before the addition of caspase 3/7 apoptosis indicator.
